# ﻿Two new species of Sordariomycetes (Chaetomiaceae and Nectriaceae) from China

**DOI:** 10.3897/mycokeys.102.114480

**Published:** 2024-03-07

**Authors:** Hai-Yan Wang, Xin Li, Chun-Bo Dong, Yan-Wei Zhang, Wan-Hao Chen, Jian-Dong Liang, Yan-Feng Han

**Affiliations:** 1 Institute of Fungus Resources, Department of Ecology, College of Life Science, Guizhou University, Guiyang 550025 Guizhou, China Guizhou University Guiyang China; 2 School of Biological Sciences, Guizhou Education University, Guiyang 550018, China Guizhou Education University Guiyang China; 3 Center for Mycomedicine Research, Basic Medical School, Guizhou University of Traditional Chinese Medicine, Guiyang 550025, Guizhou, China Guizhou University of Traditional Chinese Medicine Guiyang China

**Keywords:** Fungal taxonomy, mesophilic fungus, phylogeny, thermophilic fungus, two new taxa

## Abstract

Rich and diverse fungal species occur in different habitats on the earth. Many new taxa are being reported and described in increasing numbers with the advent of molecular phylogenetics. However, there are still a number of unknown fungi that have not yet been discovered and described. During a survey of fungal diversity in different habitats in China, we identified and proposed two new species, based on the morphology and multi-gene phylogenetic analyses. Herein, we report the descriptions, illustrations and molecular phylogeny of the two new species, *Bisifusariumkeratinophilum***sp. nov.** and *Ovatosporasinensis***sp. nov.**

## ﻿Introduction

The species diversity of fungi on earth is extremely rich, with some studies suggesting that there are as many as 5.1 million species of fungi (Blackwell 2011), while others believe that there are 3.8 million species of fungi on the earth (Hawksworth and Lücking 2017). More recent estimates suggest 2.5 million fungal species (Niskanen et al. 2023). With the rapid increase in fungal DNA sequence data obtained, the species names and numbers of fungi are constantly updated (Wijayawardene et al. 2020). fungi are one of the most diverse microbial communities on Earth and play a vital role in ecosystem processes and functions (Hyde et al. 2020). Meanwhile, fungi have an important influence on human life and production. On the one hand, they can produce a large number of biometabolites available to humans, such as various amino acids, enzymes, sugars, lipids, vitamins and antibiotics (Zhang et al. 2013; de Cassia Pereira et al. 2015; Pejin et al. 2019; Yokokawa et al. 2021; Arsenault et al. 2022; Mapook et al. 2022). On the other hand, they also infect humans, animals and plants and then cause great harm to human health and national economies (Fisher et al. 2012; Fisher et al. 2020; Zhang et al. 2023). At the same time, fungi widely exist in various habitats, such as forests, grasslands, zoos, hospitals, agricultural land (Li et al. 2014; Shao et al. 2021; Yao et al. 2021; Liu et al. 2022).

Due to factors such as global climate change, urban growth and environmental pollution, there is an increasingly accelerated loss of natural habitats worldwide, which, in turn, leads to a decrease in species diversity and the abundance of non-human organisms (Driscoll et al. 2018; Kurth et al. 2021). At present, the threat to species and their extinction rates have risen to dangerous levels threatening biological diversity. Latest data from the International Union for Conservation of Nature (IUCN) has fuelled growing societal concern, indicating that 28% of all assessed species are threatened with extinction, which is a nerve-wrackingly high figure (Löbl et al. 2023). In times of a biodiversity crisis, the community structure and species diversity of fungi are also inevitably affected by various factors. In many habitats, it is suspected that species are disappearing before they are discovered (Wang et al. 2018; Löbl et al. 2023). Therefore, it is necessary to accelerate the intensity and speed of investigating. Study on the diversity of fungal species on the earth should be one of the important issues of modern biology (Löbl et al. 2023).

Fortunately, our team has discovered many new fungal species during the investigation of fungal diversity in different habitats in China (Li et al. 2022a, b; Ren et al. 2022; Zhang et al. 2023; Wang et al. 2023). In this study, based on the morphology and multi-gene phylogenetic analyses, two new species from zoo soils were identified and described, respectively.

## ﻿Materials and methods

### ﻿Sample collection and fungal isolation

Soil samples were collected from two zoos, Shandong Province, China. Samples from 3–10 cm below the soil surface were collected, and placed in Ziploc plastic bags and brought back to the laboratory. Then, the 2 g collected samples were placed into a sterile conical flask containing 20 ml sterile water and thoroughly shaken using a Vortex vibration meter. Next, the suspension was diluted to a concentration of 10^-3^. Subsequently, 1 ml of the diluted sample was added to a sterile Petri dish and mixed with Sabouraud’s dextrose agar (SDA; peptone 10 g/l, dextrose 40 g/l, agar 20 g/l, 3.3 ml of 1% Bengal red aqueous solution) medium containing 50 mg/l penicillin and 50 mg/l streptomycin. After the plates were incubated at 25 °C and 45 °C for 1–2 weeks, single colonies were transferred from the plates to new potato dextrose agar (PDA, potato 200 g/l, dextrose 20 g/l, agar 20 g/l) plates.

### ﻿Morphological study

The target strains were transferred to plates of malt extract agar (MEA), oatmeal agar (OA) and potato dextrose agar (PDA) and were incubated at 25 °C and 45 °C. After seven days, their colony characteristics (the colony colours and diameters) on the surface and reverse of inoculated Petri dishes were observed and recorded and microscopic characteristics (fungal hyphae and conidiogenous structures) were examined and captured by making direct wet mounts with 25% lactic acid on PDA, with an optical microscope (DM4 B, Leica). The ex-types of two new species were deposited in the China General Microbiological Culture Collection Center (**CGMCC**) and living cultures and dried holotypes were deposited in the Institute of Fungus Resources, Guizhou University (**GZUIFR** = **GZAC**). Taxonomic descriptions and nomenclature of two new species were recorded in MycoBank (https://www.mycobank.org/).

### ﻿DNA extraction, PCR amplification and sequencing

Total genomic DNA was extracted using the BioTeke Fungus Genomic DNA Extraction kit (DP2032, BioTeke) following the manufacturer’s instruction. Primer combinations such as ITS1/ITS4 (White et al. 1990), LR0R/LR5 (Wang et al. 2022a), EF1-728F/EF2 (O’Donnell et al. 1998; Carbone and Kohn 1999), CAL-228F/CAL2Rd (Carbone and Kohn 1999; Lombard et al. 2015), rpb2-5F2/rpb2-7CR (Sung et al. 2007; O’Donnell et al. 2007) and T1/TUB4Rd (O’Donnell and Cigelnik 1997; Woudenberg et al. 2009) were used for amplification of the internal transcribed spacers (ITS), the 28S nrRNA locus (LSU), translation elongation factor 1-alpha gene region (*tef1*), calmodulin gene (*cmdA*), RNA polymerase II second largest subunit gene (*rpb2*) and beta-tubulin gene (*tub2*), respectively. The PCR products were sent to Quintarabio (Wuhan, China) for purification and sequencing. The new sequences were submitted to GenBank (https://www.ncbi.nlm.nih.gov/) (Table 1).

**Table 1.** Strain and GenBank accession included in phylogenetic analyses.

Species	Strains	ITS	LSU	*tef1*	*cmdA*	*rpb2*	*tub2*	Reference
*Bisifusariumaseptatum*	LC13607	MW016390	MW016390	MW580430	MW566257	MW474376	MW533717	Wang et al. (2022b)
	LC13608	MW016391	MW016391	MW580431	MW566258	MW474377	MW533718	Wang et al. (2022b)
*Bisifusariumallantoides*	UBOCC-A-120035	MW654536	MW654511	MW811075	MW811017	MW811060	MW811090	Savary et al. (2021)
	UBOCC-A-120036T	MW654548	MW654523	MW811087	MW811029	MW811072	MW811102	Savary et al. (2021)
	UBOCC-A-120037	MW654549	MW654524	MW811088	MW811030	MW811073	MW811103	Savary et al. (2021)
*Bisifusariumbiseptatum*	CBS 110311T	MW654547	MW654522	MW811086	MW811028	MW811071	MW811101	Savary et al. (2021)
*Bisifusariumdimerum*	MNHN-RF-05625T	MW654546	MW654521	MW811085	MW811027	–	MW811100	Savary et al. (2021)
	CBS 108944T	JQ434586	JQ434514	KR673912	KM231365	KM232363	EU926400	Lombard et al. (2015)
*Bisifusariumpenicilloides*	UBOCC-A-120021T	MW654542	MW654517	MW811081	MW811023	MW811066	MW811096	Savary et al. (2021)
	UBOCC-A-120034	MW654541	MW654516	MW811080	MW811022	MW811065	MW811095	Savary et al. (2021)
	VTT-D-041022	MW654535	MW654510	MW811074	MW811016	MW811059	MW811089	Savary et al. (2021)
*Bisifusariumdelphinoides*	CBS 120718T	EU926229	EU926229	EU926296	KM231363	–	EU926362	Lombard et al. (2015)
	CBS 110140	MW827603	–	EU926302	–	–	EU926368	Park et al. (2019)
	CBS 110310	EU926240	EU926240	EU926307	–	–	EU926373	Sun et al. (2017)
*Bisifusariumnectrioides*	CBS 176.31T	EU926245	EU926245	EU926312	KM231362	–	EU926378	Lombard et al. (2015)
*Bisifusariumpenzigii*	CBS 116508	EU926256	EU926256	EU926323	–	–	EU926389	Sun et al. (2017)
*Bisifusariumdomesticum*	CBS 102407	EU926221	EU926221	EU926288	–	–	EU926355	Sun et al. (2017)
	CBS 244.82	EU926220	EU926220	EU926287	–	–	EU926354	Sun et al. (2017)
*Bisifusariumlunatum*	CBS 632.76T	EU926224	EU926224	EU926291	KM231367	–	EU926357	Lombard et al. (2015)
*Bisifusariumtonghuanum*	CGMCC3.17369	KX790413	KX790414	KX790418	–	–	KX790417	Sun et al. (2017)
	CGMCC3.17370	KX790415	KX790416	KX790420	–	–	KX790419	Sun et al. (2017)
*Bisifusariumlovelliae*	BRIP 75047a	OQ629340	–	–	–	OQ626864	–	Tan et al. (2023)
***Bisifusariumkeratinophilum***	**CGMCC 3.23621**T	**OP693473**	**OP693469**	OR168082	OR043998	**OR168079**	**OR168085**	**This study**
	**GZUIFR 22.371**	**OP693474**	**OP693470**	**OR168083**	**OR043999**	**OR168080**	**OR168086**	**This study**
	**GZUIFR 22.372**	**OP693475**	**OP693471**	**OR168084**	**OR044000**	**OR168081**	**OR168087**	**This study**
*Longinectrialagenoides*	UBOCC-A-120039	MW654539	MW654514	MW811078	MW811020	MW811063	MW811093	Savary et al. (2021)
*Longinectriaverticilliforme*	UBOCC-A-120043	MW654540	MW654515	MW811079	MW811021	MW811064	MW811094	Savary et al. (2021)
*Ovatosporaamygdalispora*	CBS 672.82T	–	–	–	–	MZ342991	MZ343030	Wang et al. (2022a)
*Ovatosporaangularis*	LC3973	KP336768	KP336817	–	–	KT149491	KP336866	Wang et al. (2022a)
*Ovatosporaunipora*	CBS 109.83T	KX976689	KX976787	–	–	KX976902	KX977037	Wang et al. (2016)
*Ovatosporabrasiliensis*	CBS 140.50	KX976683	KX976781	–	–	KX976896	KX977031	Wang et al. (2016)
*Ovatosporamedusarum*	CBS 148.67T	KX976684	KX976782	–	–	KX976897	KX977032	Wang et al. (2016)
*Ovatosporamollicella*	CBS 583.83T	KX976685	KX976783	–	–	KX976898	KX977033	Wang et al. (2016)
*Ovatosporapseudomollicella*	CBS 251.75T	KX976686	KX976784	–	–	KX976899	KX977034	Wang et al. (2016)
*Ovatosporasenegalensis*	CBS 728.84T	KX976687	KX976785	–	–	KX976900	KX977035	Wang et al. (2016)
*Trichocladiumasperum*	CBS 903.85T	LT993632	LT993632	–	–	LT993551	LT993713	Wang et al. (2022a)
*Trichocladiumacropullum*	CBS 114580T	LT993626	LT993626	–	–	LT993545	LT993707	Wang et al. (2022a)
*Trichocladiumamorphum*	CBS 127763T	LT993628	LT993628	–	–	LT993547	LT993709	Wang et al. (2022a)
*Trichocladiumantarcticum*	CBS 123565T	LT993629	LT993629	–	–	LT993548	LT993710	Wang et al. (2022a)
*Trichocladiumbeniowskiae*	CBS 757.74T	LT993635	LT993635	–	–	LT993554	LT993716	Wang et al. (2022a)
*Trichocladiumgilmaniellae*	CBS 388.75T	LT993638	LT993638	–	–	LT993557	LT993719	Wang et al. (2022a)
*Thermochaetoidesdissita*	CBS 180.67T	–	MK919319	–	–	MK919375	MK919433	Wang et al. (2022a)
*Thermochaetoidesthermophila*	CBS 144.50T	–	MK919314	–	–	KM655436	MK919428	Wang et al. (2022a)
***Ovatosporasinensis***	**CGMCC40675**T	**OR016676**	**OR016679**	–	–	**OR043992**	**OR043995**	**This study**
	**GZUIFR 23.002**	**OR016677**	**OR016680**	–	–	**OR043993**	**OR043996**	**This study**
	**GZUIFR 23.003**	**OR016678**	**OR016681**	–	–	**OR043994**	**OR043997**	**This study**
*Triangulariaverruculos*a	CBS 148.77	MK926874	MK926874	–	–	MK876836	MK926974	Wang et al. (2022a)
*Triangulariaallahabadensis*	CBS 724.68T	MK926865	MK926865	–	–	MK876827	MK926965	Wang et al. (2022a)

Note: T=Ex-type; New isolates are in bold; The line “–” represents the absence of GenBank record; BRIP: Queensland Plant Pathology Herbarium, Australia; CBS: CBS-KNAW Fungal Biodiversity Centre, Utrecht, The Netherlands; CGMCC: The China General Microbiological Culture Collection Centre; GZUIFR: The Institute of Fungus Resources, Guizhou University, China; LC: Lei Cai’s personal culture collection, Beijing, China; MNHN: Museum National d’Histoire Naturelle culture collection, France; UBOCC: Universitée de Bretagne Occidentale Culture Collection, France; VTT: Culture Collection, Finland; *cmdA*: calmodulin; ITS: the internal transcribed spacer region and intervening 5.8S nrRNA; LSU: 28S large subunit; *rpb2*: RNA polymerase II second largest subunit; *tef1*: translation elongation factor 1-alpha; *tub2*: β-tubulin.

### ﻿Phylogenetic analysis

In this study, the relevant sequences were obtained from GenBank (Table 1). The sequence set was aligned and trimmed in MEGA v.6.06 (Tamura et al. 2013). We performed single gene and multi-gene phylogenetic analysis using ITS, LSU, *tef1*, *cmdA*, *rpb2* and *tub2* gene and found that the topology structures of the single-gene and multi-gene phylogenetic trees were consistent in PhyloSuite v.1.16. Therefore, multi-gene phylogenetic analysis was chosen in this study. The concatenation of loci and phylogenetic analysis were processed, using the “Concatenate Sequence” function in PhyloSuite v.1.16 (Zhang et al. 2020). The Maximum Likelihood (ML) and the Bayesian Inference (BI) methods were used for the phylogenetic construction of each loci dataset. The ML analysis was conducted in IQ-TREE v.1.6.11 (Nguyen et al. 2015) with 1000 bootstrap tests using the ultrafast algorithm (Minh et al. 2013). The BI analysis was performed in MrBayes v.3.2 (Ronquist et al. 2012) and Markov chain Monte Carlo (MCMC) simulations were used for 2,000,000 generations with a sampling frequency of every 100 generations. The phylogenetic trees were visualised using FigTree version 1.4.3 and subsequently edited in Adobe Photoshop.

## ﻿Results

### ﻿Phylogenetic analysis

The ITS regions of all isolates were sequenced and BLASTn searched in NCBI. Our isolates were identified as two genera, *Bisifusarium* L. Lombard, Crous & W. Gams and *Ovatospora* X.Wei Wang, Samson & Crous, respectively. The ITS sequences of the isolated strains were less than 97% similarity to the closest strains in GenBank and were considered as the potential new species.

**Figure 1.**
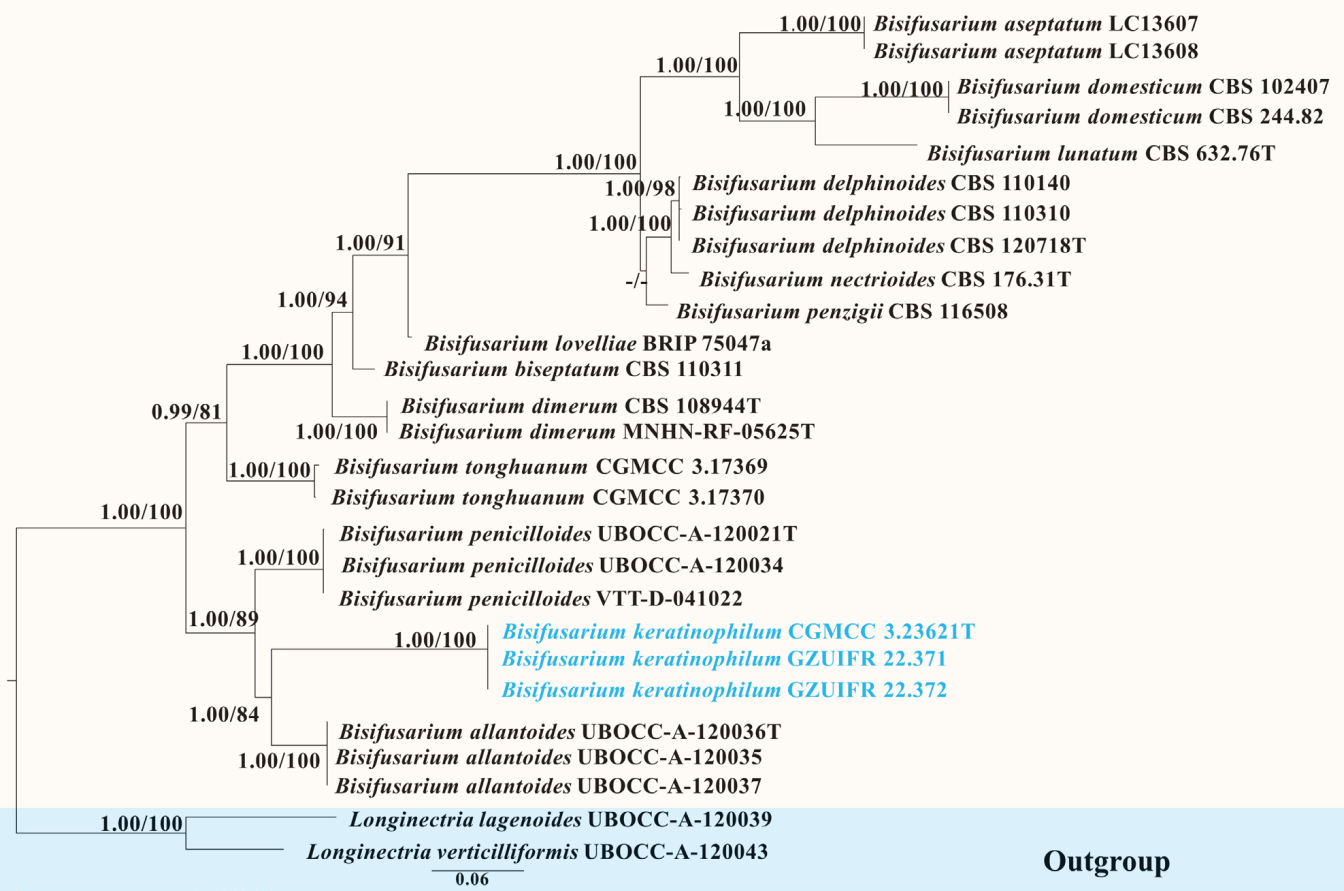
Phylogenetic tree of the genus *Bisifusarium* constructed from the dataset of ITS, LSU, *tef1*, *cmdA*, *rpb2* and *tub2*. Notes: Statistical support values (BI/ML) were shown at nodes. ML bootstrap values ≥ 75% and posterior probabilities ≥ 0.90 are shown above the internal branches. ‘–’ indicates the absence of statistical support (< 75% for bootstrap proportions from ML analysis; < 0.90 for posterior probabilities from Bayesian analysis). Three new strains are shown in blue font. BRIP: Queensland Plant Pathology Herbarium, Australia; CBS: CBS-KNAW Fungal Biodiversity Centre, Utrecht, The Netherlands; CGMCC: The China General Microbiological Culture Collection Centre; GZUIFR: The Institute of Fungus Resources, Guizhou University, China; LC: Lei Cai’s personal culture collection, Beijing, China; MNHN: Museum National d’Histoire Naturelle culture collection, France; UBOCC: Universitée de Bretagne Occidentale Culture Collection, France; VTT: Culture Collection, Finland.

To further determine the phylogenetic position of these isolated strains, we performed a multi-locus phylogenetic analysis, based on ITS, LSU, *tef1*, *cmdA*, *rpb2* and *tub2* gene. The phylogenetic trees (Figs 1, 3) using ML and BI analyses were consistent and strongly supported in most branches. The ML analysis for the combined dataset provided the best scoring tree. The best-fit evolutionary models for ML analysis and BI analysis are shown in Table 2.

**Table 2.** The best-fit evolutionary models.

Genus		ITS	LSU	*tef1*	*cmdA*	*rpb2*	*tub2*
*Bisifusarium*	ML analysis BI analysis	TIM2e+I+G4 SYM+I+G4	K2P K2P	TNe+R2 K2P+G4	TIM3e+I+G4 SYM+I+G4	TIM3e+I+G4 SYM+I+G4	TIM3e+I+G4 SYM+I+G4
*Ovatospora*	ML analysis BI analysis	GTR+F+G4 GTR+F+G4	TIM3+F+I GTR+F+I			TIM3+F+G4 GTR+F+I+G4	HKY+F+I+G4 HKY+F+I+G4

In this study, three isolates of the genus *Bisifusarium* clustered in a well-separated clade with a high support value (BI/ML 1/100) (Fig. 1). Three isolates of the genus *Ovatospora* clustered together with a high support value (BI/ML 1/100) (Fig. 3). Therefore, *Bisifusariumkeratinophilum* H.Y. Wang, X. Li & Y.F. Han, sp. nov. and *Ovatosporasinensis* H.Y. Wang & Y.F. Han, sp. nov. are proposed according to the phylogenetic analysis.

## ﻿Taxonomy

### ﻿Sordariomycetes O.E. Erikss. & Winka

**Hypocreales Lindau**

**Nectriaceae Tul. & C. Tul.**

***Bisifusarium* L. Lombard, Crous & W. Gams**

#### ﻿ Bisifusarium keratinophilum

Taxon classificationFungiHypocrealesNectriaceae

H.Y. Wang, X. Li & Y.F. Han sp. nov.

A26A1774-6937-5017-8D31-6CBA1E0AB0A8

MycoBank No: 849504

Fig. 2

##### Etymology.

Referring to degradation properties of chicken feathers.

##### Type.

China: Shandong Province, Jinan City, Jinan Zoo (36°42'14"N, 116°58'55"E), soil, July 2021, Xin Li & Yan-Feng Han, ex-type CGMCC 3.23621 = GZUIFR 22.370, dried holotype GZAC 22.370.

**Figure 2.**
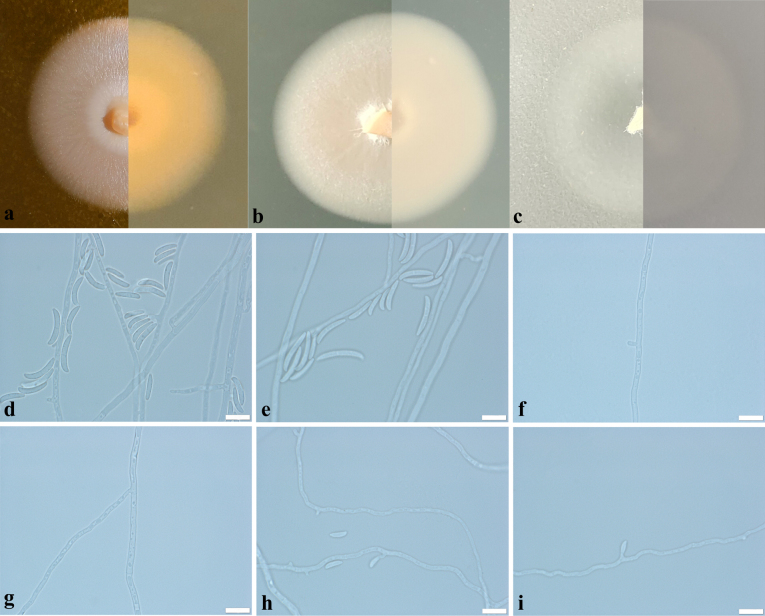
Morphological characteristics of *Bisifusariumkeratinophilum* sp. nov. **a–c** front and reverse of colony on MEA, OA and PDA after 7 days at 25 °C **d, e** conidiophores and macroconidia **f** phialidic pegs **g** hyphae **h, i** microconidia. Scale bars: 10 μm (**d–i**).

**Figure 3.**
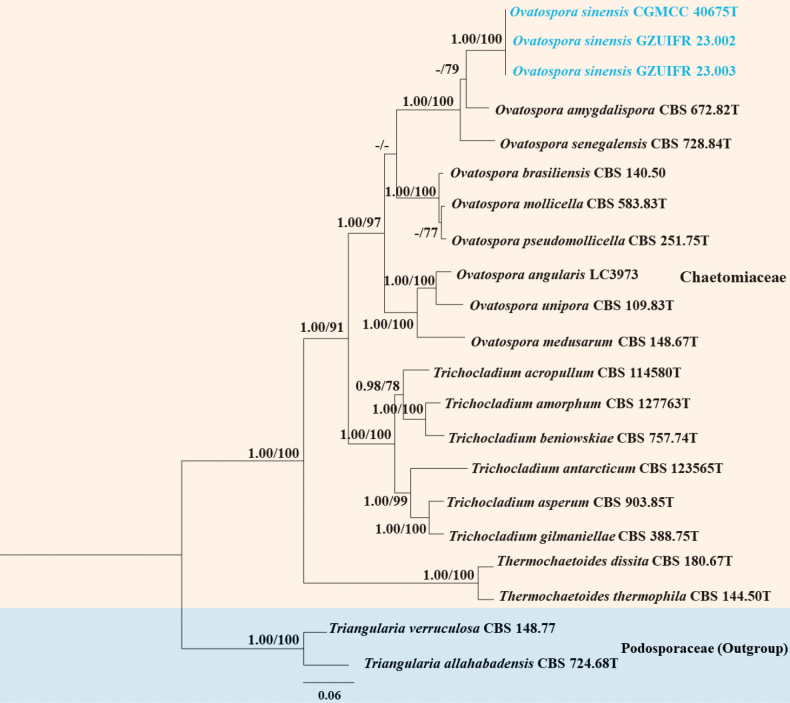
Phylogenetic tree of the genus *Ovatospora* constructed from ITS, LSU, *tub2* and *rpb2*. Notes: Statistical support values (BI/ML) were shown at nodes. ML bootstrap values ≥ 75% and posterior probabilities ≥ 0.90 are shown above the internal branches. ‘–’ indicates the absence of statistical support (< 75% for bootstrap proportions from ML analysis; < 0.90 for posterior probabilities from Bayesian analysis). Three new strains are shown in blue. CBS: CBS-KNAW Fungal Biodiversity Centre, Utrecht, The Netherlands; CGMCC: The China General Microbiological Culture Collection Centre; GZUIFR: The Institute of Fungus Resources, Guizhou University, China; LC: Lei Cai’s personal culture collection, Beijing, China.

##### Description.

Culture characteristics: Colonies growing on MEA, OA and PDA after 7 days of incubation at 25 °C. On MEA, reaching up 20–25 mm diam., thick villiform, cream (RAL9001) at the centre, oyster white (RAL1013) at the edge, mostly regular in the margin, reverse light ivory (RAL 1015); On OA, reaching up 25–35 mm diam.; pure white (RAL9010), thin, villiform, mostly regular in the margin, reverse tele grey 4 (RAL7047); On PDA, reaching up 25–30 mm diam.; cream (RAL9001), thin, short villiform, mostly regular in the margin, reverse cream (RAL9001).

On PDA medium, ***Hyphae*** septate, hyaline, smooth, thick-walled, 1.5–3.5 μm wide. ***Conidiophores*** arising from hyphae, solitary, smooth, mostly clavate, 5–25 × 1–2.5 μm. Phialidic pegs arising from hyphae. ***Monophialides*** laterally on hyphae or phialidic pegs, cylindrical, erect. ***Polyphialides*** absent. ***Macroconidia*** produced by monophialidic conidiophores, mostly 0-1septate, rarely 2-septate, mostly crescent, rarely clavate, 12–23.0 × 2.0–3.5 μm (av. 16 × 2.5 μm, n = 50). ***Microconidia*** produced by later phialidic pegs, monocelled, cymbiform, 6.0–9.5 × 1.5–2.5 μm (av. 7.5 × 2.0 μm, n = 50).

##### Additional materials examined.

China: Shandong Province, Jinan City, Jinan Zoo (36°42'14"N, 116°58'55"E), soil, July 2021, living cultures GZUIFR 22.371, GZUIFR 22.372.

##### Notes.

Phylogenetically, our three strains (CGMCC 3.23621, GZUIFR 22.371 and GZUIFR 22.372) of *Bisifusariumkeratinophilum* H.Y. Wang, X. Li & Y.F. Han sp. nov. clustered in a single separate clade with a high support value (BI/ML 1/100). Although it was closely related to *B.allantoides* O. Savary, M. Coton, E. Coton & J.L. Jany and *B.penicilloides* O. Savary, M. Coton, E. Coton & J.L. Jany in the phylogenetic tree, *B.allantoides* had allantoidal macroconidia (Savary et al. 2021) and *B.penicilloides* had ellipsoidal and reniform macroconidia and absent microconidia (Savary et al. 2021). *Bisifusariumkeratinophilum* can be distinguished from the other previously described species by having crescent and clavate macroconidia and cymbiform microconidia.

Our team found that *B.keratinophilum* has the ability to degrade chicken feathers. Specific method: the spore suspension (10^7^spores per millilitre) was inoculated into the fermentation medium containing 1g chicken feathers and cultured in a shaking table at 150 rpm, 30 °C for 96 h, then the chicken feather residue was filtered, dried and weighed. This fungus had a good degradation effect on chicken feathers with the degradation rate of 52.02%.

### ﻿Sordariomycetes O.E. Erikss. & Winka

**Sordariales Chadef. ex D. Hawksw. & O.E. Erikss.**

**Chaetomiaceae G. Winter**

***Ovatospora* X.Wei Wang, Samson & Crous**

#### ﻿ Ovatospora sinensis

Taxon classificationFungiSordarialesChaetomiaceae

H.Y. Wang &Y.F. Han sp. nov.

A63A0DBB-63D9-5192-89CA-EE3AE53019F4

MycoBank No: 850259

Fig. 4

##### Etymology.

Refers to China where the species was discovered.

##### Type.

China: Shandong Province, Qingdao City, Qingdao Zoo (35°59'14"N, 120°3'53"E), soil, July 2021, Hai-Yan Wang & Yan-Feng Han, ex-type CGMCC 40675=GZUIFR 23. 001, dried holotype GZAC 23. 001.

**Figure 4.**
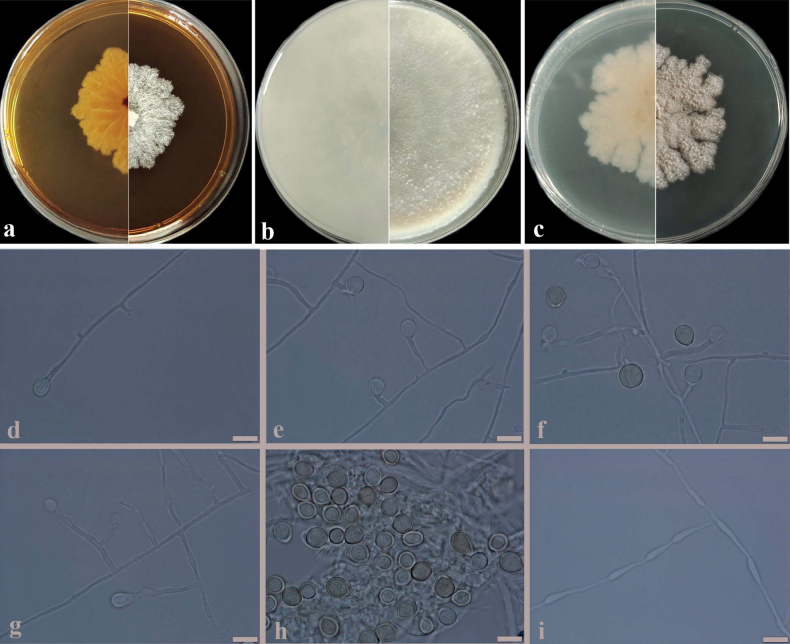
Morphological characteristics of *Ovatosporasinensis* sp. nov. **a–c** reverse and front of colony on MEA, OA and PDA after7 days at 45 °C **d–h** conidiophores and conidia **i** hyphae. Scale bars: 10 μm (**d–i**).

##### Description.

Culture characteristics: Colonies growing on MEA, OA and PDA after 7 days of incubation at 45 °C. Colony on MEA reaching about 35–45 mm diam., pure white (RAL9010), densely villiform; irregular in the margin; reverse light ivory (RAL1015), radial lines, irregular in the margin. Colony on OA reaching about 80–90 mm diam., grey white (RAL9002), sparsely aerial mycelium, mostly regular in the margin; reverse grey white (RAL9002). Colony on PDA reaching about 45–50 mm diam., creamy (RAL9001), densely villiform obviously powdery conidia group, sparsely spongy, irregular in the margin; reverse creamy (RAL9001), plicated at the centre, irregular in the margin.

***Hyphae*** septate, hyaline, smooth, thin-walled, 1.5–3.5 μm wide. ***Conidiophores*** arising from hyphae, 2–30 × 1.5–3.5 μm, solitary or branched, smooth, mostly clavate, septate. ***Conidiogenous cell*** reduced to Conidiophores. ***Conidia*** on conidiogenous or acrogenous directly on the hyphae, hyaline or light-brown, mostly globose, rarely obovate, thick-walled, 6.0–10.5 μm diam. (av. 8.0 μm). Sexual morph unknown.

##### Additional specimens examined.

China. Shandong Province, Qingdao City, Qingdao Zoo (35°59'14"N, 120°3'53"E), soil, July 2021, Hai-Yan Wang & Yan-Yeng Han, living cultures GZUIFR 23.002, GZUIFR 23.003.

##### Notes.

Phylogenetically, our three strains (CGMCC 40675, GZUIFR 23.002 and GZUIFR 23.003) of *Ovatosporasinensis* H.Y. Wang &Y.F. Han sp. nov. clustered together in a single clade with a high support value (BI/ML 1/100). Although it was closely related to *O.amygdalispora* (Udagawa & T. Muroi) X.Wei Wang & Houbraken and *O.senegalensis* (Ames) X. Wei Wang & Samson, it has an apparent separate subclade. Morphologically, *O.amygdalispora* and *O.senegalensis* only have the sexual structures, while *Ovatosporasinensis* sp. nov. only produce an asexual morph with clavate and solitary or ramiform conidiophores and globose conidia. So far, *Ovatosporasinensis* sp. nov. is the only species that produces an asexual morph and is a thermophilic fungus in the genus *Ovatospora*.

## ﻿Discussion

Lombard et al. (2015) re-estimated the status of those genera lacking DNA sequence data in Nectriaceae, based on the morphology and multi-gene phylogenetic analyses and the new genus *Bisifusarium* with the type *B.dimerum* (Penz.) L. Lombard & Crous was proposed, which formed a well-supported clade (ML = 100%, BYPP = 1.0) and separated from the clade of Fusarium. Therefore, these fusarium-like species including *B.biseptatum* (Schroers, Summerbell & O’Donnell) L. Lombard & Crous, *B.delphinoides* (Schroers, Summerbell, O’Donnell & Lampr.) L. Lombard & Crous, *B.dimerum*, *B.domesticum* (Fr.) L. Lombard & Crous, *B.lunatum* (Ellis & Everh.) L. Lombard & Crous, *B.nectrioides* (Wollenw.) L. Lombard & Crous Schroers, Summerbell & O’Donnell) and *B.penzigii* (Schroers, Summerbell & O’Donnell) L. Lombard & Crous, were transferred from the genus *Fusarium* Link to this new genus *Bisifusarium*. The genus *Bisifusarium* produces macroconidia below three septa and forms lateral phialidic pegs arising from the hyphae, which can be distinguished from the other species in the genus *Fusarium* (Schroers et al. 2009; Lombard et al. 2015). Recently, several new species in genus *Bisifusarium* have been published. Presently, *Bisifusarium* contains fifteen species records in the Index Fungorum (http://www.indexfungorum.org/Names/Names.asp, retrieval on 18 October 2023). Here, excluding synonyms and adding *B.keratinophilum* sp. nov., the genus *Bisifusarium* has a total of fourteen species.

Based on the morphology and phylogenetic analysis of a combined dataset of ITS, LSU, *rpb2*and *tub2* sequence data, Wang et al. (2016) redefined the generic concept of *Chaetomium* Kunze and *Ovatospora* X. Wei Wang, Samson & Crous with the type *O.brasiliensis* (Batista & Pontual) X. Wei Wang & Samson was proposed, which formed a well-supported clade and separated from the Chaetomium clade. Therefore, these chaetomium-like species included *O.brasiliensis* (Batista & Pontual) X. Wei Wang & Samson, *O.medusarum* (Meyer & Lanneau) X. Wei Wang & Samson, *O.mollicella* (Ames) X. Wei Wang & Samson, *O.senegalensis* (Ames) X. Wei Wang & Samson and *O.unipora* (Aue & Müller) X. Wei Wang & Samson. Simultaneously, *O.pseudomollicella* X. Wei Wang & Samson sp. nov. was introduced. In addition, based on the results of the phylogeny and molecular data analyses, two new combinations, *O.amygdalispora* (Udagawa & T. Muroi) X.Wei Wang & Houbraken and *O.angularis* (Yu Zhang & L. Cai) X.Wei Wang & Houbraken from *Chaetomium* were proposed by Wang et al. (2022a). As of October 2023, the genus *Ovatospora* contains nine species: *O.amygdalispora*, *O.angularis*, *O.brasiliensis*, *O.medusarum*, *O.mollicella*, *O.pseudomollicella*, *O.senegalensis*, *Ovatosporasinensis* and *O.unipora*.

## Supplementary Material

XML Treatment for Bisifusarium keratinophilum

XML Treatment for Ovatospora sinensis

## ﻿Additional information

### Author ORCIDs

Hai-Yan Wang https://orcid.org/0000-0001-9190-0490

Xin Li https://orcid.org/0000-0001-7910-1469

Chun-Bo Dong https://orcid.org/0000-0001-7074-5700

Yan-Wei Zhang https://orcid.org/0000-0003-1251-5821

Wan-Hao Chen https://orcid.org/0000-0001-7240-6841

Jian-Dong Liang https://orcid.org/0000-0002-3939-3900

Yan-Feng Han https://orcid.org/0000-0002-8646-3975

### Data availability

All of the data that support the findings of this study are available in the main text or Supplementary Information.

## Supplementary materials

Supplementary material 1 The alignments used in the phylogenetic analysis Data type zip https://binary.pensoft.net/file/998100This dataset is made available under the Open Database License (http://opendatacommons.org/licenses/odbl/1.0/). The Open Database License (ODbL) is a license agreement intended to allow users to freely share, modify, and use this Dataset while maintaining this same freedom for others, provided that the original source and author(s) are credited.Hai-Yan Wang, Yan-Feng Han
